# A selective inhibitor of histone deacetylase 3 prevents cognitive deficits and suppresses striatal CAG repeat expansions in Huntington’s disease mice

**DOI:** 10.1038/s41598-017-05125-2

**Published:** 2017-07-20

**Authors:** Nuria Suelves, Lucy Kirkham-McCarthy, Robert S. Lahue, Silvia Ginés

**Affiliations:** 1 Departament de Biomedicina, Facultat de Medicina, Universitat de Barcelona, Barcelona, Spain; 2 Institut d’ Investigacions Biomèdiques August Pi i Sunyer (IDIBAPS), Barcelona, Spain; 3 Centro de Investigación Biomédica en Red sobre Enfermedades Neurodegenerativas (CIBERNED), Madrid, Spain; 4 Institut de Neurociències, Universitat de Barcelona, Barcelona, Spain; 5 Centre for Chromosome Biology, National University of Ireland Galway, Newcastle Road, Galway, Ireland; 6 NCBES Galway Neuroscience Centre, National University of Ireland Galway, Newcastle Road, Galway, Ireland

## Abstract

Huntington’s disease (HD) is a neurodegenerative disorder whose major symptoms include progressive motor and cognitive dysfunction. Cognitive decline is a critical quality of life concern for HD patients and families. The enzyme histone deacetylase 3 (HDAC3) appears to be important in HD pathology by negatively regulating genes involved in cognitive functions. Furthermore, HDAC3 has been implicated in the aberrant transcriptional patterns that help cause disease symptoms in HD mice. HDAC3 also helps fuel CAG repeat expansions in human cells, suggesting that HDAC3 may power striatal expansions in the *HTT* gene thought to drive disease progression. This multifaceted role suggests that early HDAC3 inhibition offers an attractive mechanism to prevent HD cognitive decline and to suppress striatal expansions. This hypothesis was investigated by treating Hdh^Q111^ knock-in mice with the HDAC3-selective inhibitor RGFP966. Chronic early treatment prevented long-term memory impairments and normalized specific memory-related gene expression in hippocampus. Additionally, RGFP966 prevented corticostriatal-dependent motor learning deficits, significantly suppressed striatal CAG repeat expansions, partially rescued striatal protein marker expression and reduced accumulation of mutant huntingtin oligomeric forms. These novel results highlight RGFP966 as an appealing multiple-benefit therapy in HD that concurrently prevents cognitive decline and suppresses striatal CAG repeat expansions.

## Introduction

Huntington’s disease (HD) is a fatal neurodegenerative disorder marked by progressive motor dysfunction, cognitive deficits and psychiatric disturbances. Currently available treatments help manage some of the symptoms but there is no cure, nor has disease progress be reversed or slowed. HD is caused by inheritance of an expanded CAG repeat in the huntingtin (*HTT)* gene, resulting in a mutant huntingtin (mHtt) protein containing extra glutamine residues^1, 2^. Increasing evidence suggests that extending the glutamine tract confers mHtt with new toxic properties, including aberrant interactions with proteins necessary for chromatin maintenance and gene expression. In doing so, mHtt may induce transcriptional dysregulation and negatively impact expression of key genes for brain activity^3, 4^. For example, the histone acetyltransferase CBP/p300 forms aggregates with mHtt, reducing acetyltransferase activity. In HD patients and mice, chromatin becomes hypoacetylated and transcriptional dysregulation occurs in many genes, including those involved in long-term memory^5–7^.

Inhibition of histone deacetylases (HDACs) is predicted to increase histone acetylation and restore normal transcriptional patterns. Recent studies used inhibitors selective for HDAC isotypes to minimize toxicity. Treatment with inhibitors primarily targeting HDAC3, including some with additional activity on HDAC1, led to improvement in motor function and working memory in N-terminal transgenic HD mice^8–12^. These benefits were correlated with attenuation of striatal atrophy, reestablishment of normal striatal and cortical gene expression patterns and modulation of epigenetic DNA modifications. These encouraging findings are consistent with the discovery that wild type but not mHtt interacts with HDAC3 and represses its neurotoxic activity^13^.

There is a gap in our understanding of potential benefits of HDAC3-selective inhibitors on cognitive functions, such as motor learning and long-term memory. HD patients and mice show significant declines in cognitive function before onset of motor symptoms^14–17^. Cognitive decline is a critical quality-of-life concern for HD patients and families^17^. Notably, HDAC3 is a negative regulator of gene expression required for long-term memory formation^18^ and HDAC3 focal deletion or selective inhibition improves memory and neural plasticity in rodents^18–22^. Together, these findings highlight HDAC3 as a key player in enforcing transcriptional dysregulation that underlies cognitive symptoms of HD. In addition, HDAC3 also stimulates expansions of CAG repeats in human tissue culture cells^23, 24^. HDAC3 inhibition or knockdown suppressed most CAG repeat expansions. In contrast, inhibition of HDAC1/HDAC2 or knockdown of HDAC1 failed to suppress expansions in tissue culture^23^. There is significant evidence that the *HTT* CAG repeat undergoes progressive tissue- and cell type-specific expansions^25–32^, which could contribute to disease progression and age of onset^33–37^. Therefore HDAC3 is also an attractive therapeutic target for inhibiting striatal expansions and delaying HD.

This study tested the idea that HDAC3 is important in HD pathology via its transcriptional repressive effects contributing to cognitive impairment and in fueling somatic CAG repeat expansions. We demonstrate that early intervention in Hdh^Q7/Q111^ knock-in mice with RGFP966, a selective HDAC3 inhibitor with some activity on HDAC1/2, prevents motor learning and long-term memory deficits, reduces striatal CAG repeat expansions and improves hippocampal and striatal pathologies. These new findings, combined with beneficial effects of HDAC3 inhibition on motor function^8–12^, indicate that RGFP966 simultaneously delivers multiple benefits in HD mice.

## Results

### Systemic RGFP966 administration efficiently inhibits HDAC activity in hippocampus and striatum

The major goal of the project was to use an HDAC3-selective inhibitor in an early-intervention strategy to prevent or reduce key HD symptoms in mice. We focused on two important aspects of HD that have not been previously tested with HDAC3 inhibitors: preventing cognitive deficits, both in motor learning and long-term memory, and suppressing somatic CAG repeat expansions. RGFP966 was chosen as the inhibitor, as it is 30- to 200-fold selective for HDAC3, with reported IC_50_ values of 64–80 nM^12, 19^. RGFP966 reaches the brain rapidly after systemic injection^19, 20^. The Hdh^Q7/Q111^ knock-in (KI) mouse was utilized because, like most HD patients, these mice are heterozygous for the *HTT* mutation, expressing one normal mouse (Q7) and one expanded (Q111) allele. Importantly, the animals reproduce key features of human disease, including an accurate expression of huntingtin protein and a similar timing of emotional, cognitive and motor impairment^38–43^. The slow progression of HD pathology provides a suitable age window for early intervention with the HDAC3 inhibitor. Additionally, since these mice carry the human HD mutation, they also provide an ideal model in which to study the instability of the HD CAG repeat in its appropriate genomic context.

**Figure 1:**
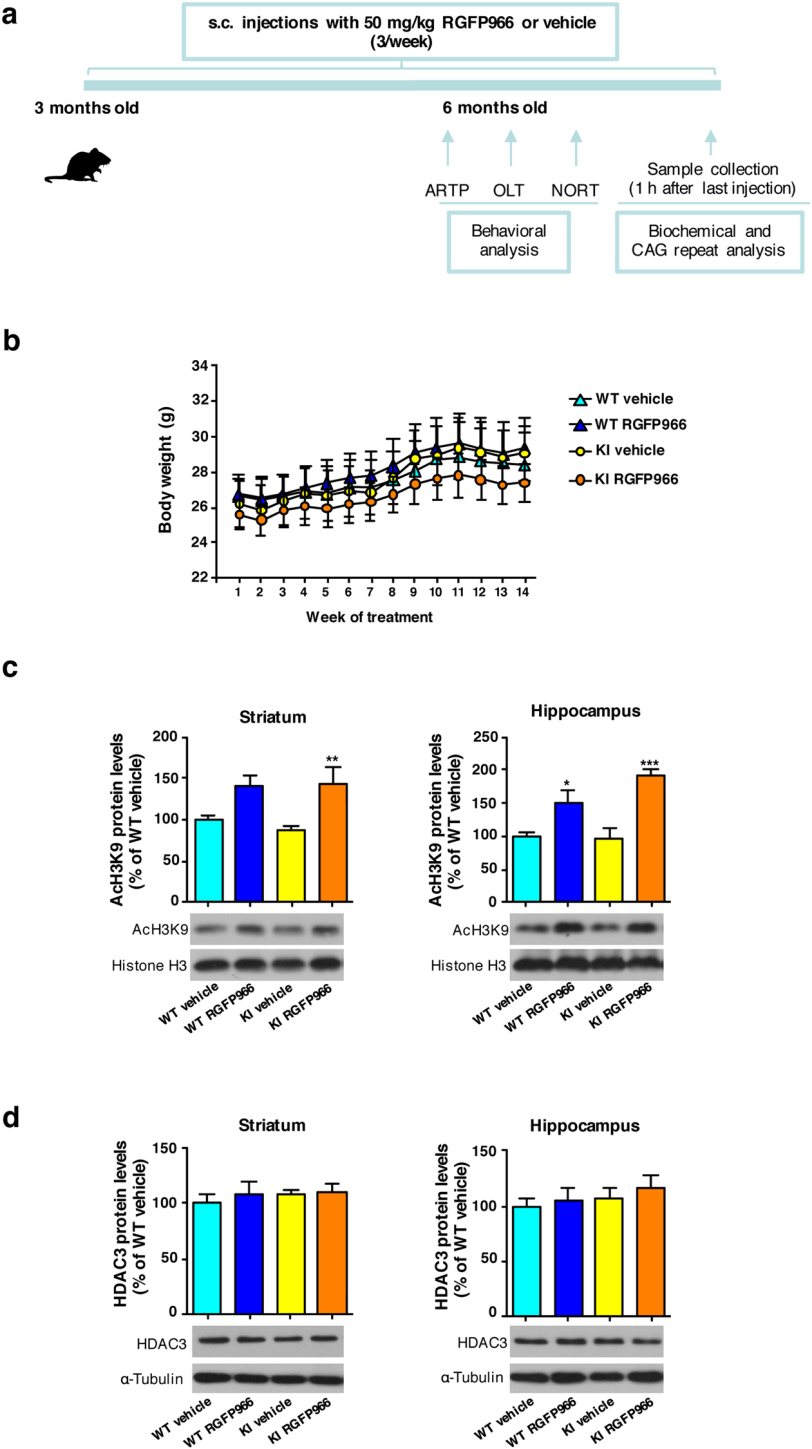
Systemic RGFP966 treatment efficiently inhibits hippocampal and striatal HDAC activity. **(a)** Schematic of chronic treatment and experimental plan. ARTP, accelerating rotarod task procedure; OLT, object location task; NORT, novel object recognition task. (**b)** Body weight measurements. Data represent the mean ± SEM (n = 6–10). (**c)** Representative immunoblots showing histone H3 acetylation levels at position lysine 9 (AcH3K9) normalized to total histone H3 and **(d)** HDAC3 levels normalized to α-tubulin in striatal and hippocampal extracts from vehicle and RGFP966 chronically treated wild type Hdh^Q7/Q7^ (WT) and mutant Hdh^Q7/Q111^ (KI) mice. The blots in **(c)** and **(d)** are cropped; full-length images are provided in Supplementary Figure 2. *p < 0.05; **p < 0.01; ***p < 0.001 compared to vehicle-treated mice by two-way ANOVA with Bonferroni *post-hoc* analysis. Data represent the mean ± SEM (n = 4–7 per group).

RGFP966 was administered subcutaneously to wild type (Hdh^Q7/Q7^) and KI (Hdh^Q7/Q111^) mice three times per week for 13 weeks starting at three months of age, prior to development of cognitive defects (Fig. 1a). The inhibitor was well tolerated, with no significant alteration in body weight compared to vehicle-treated animals (Fig. 1b). Mice were consequently analyzed for behavior at six months of age when cognitive but not motor symptoms start to appear (Fig. 1a). All animals were sacrificed subsequently and key brain tissues were examined by biochemical and genetic analysis.

Cognitive behavioral tasks described in the next section are mostly dependent on the hippocampus and striatum, so it was important to demonstrate drug activity in these tissues. Examination of histone H3 acetylation at lysine position 9 (AcH3K9) in hippocampal and striatal regions showed drug-dependent increases in both wild type and KI animals (Fig. 1c), consistent with a decrease in HDAC3 activity. Reduced HDAC3 activity was likely due to inhibition of enzyme activity, since the abundance of HDAC3 protein was unchanged between genotypes or by RGFP966 treatment (Fig. 1d). These findings indicate efficacy of systemic administration of RGFP966 in inhibiting HDAC activity in the hippocampus and striatum.

### RGFP966 prevents hippocampal-dependent long-term memory deficits

Cognitive dysfunction is an early clinical hallmark of HD that precedes motor coordination deficits, both in KI mice^39, 40^ and in HD patients^15–17^. Even though it is known that HDAC3 acts as a negative regulator of gene expression required for long-term memory formation^18^, no data have been reported on the effect of HDAC3 inhibition on long-term memory impairments in HD. Therefore, we investigated whether RGFP966 treatment could block or delay impairments in hippocampal-dependent recognition and spatial memories.

**Figure 2:**
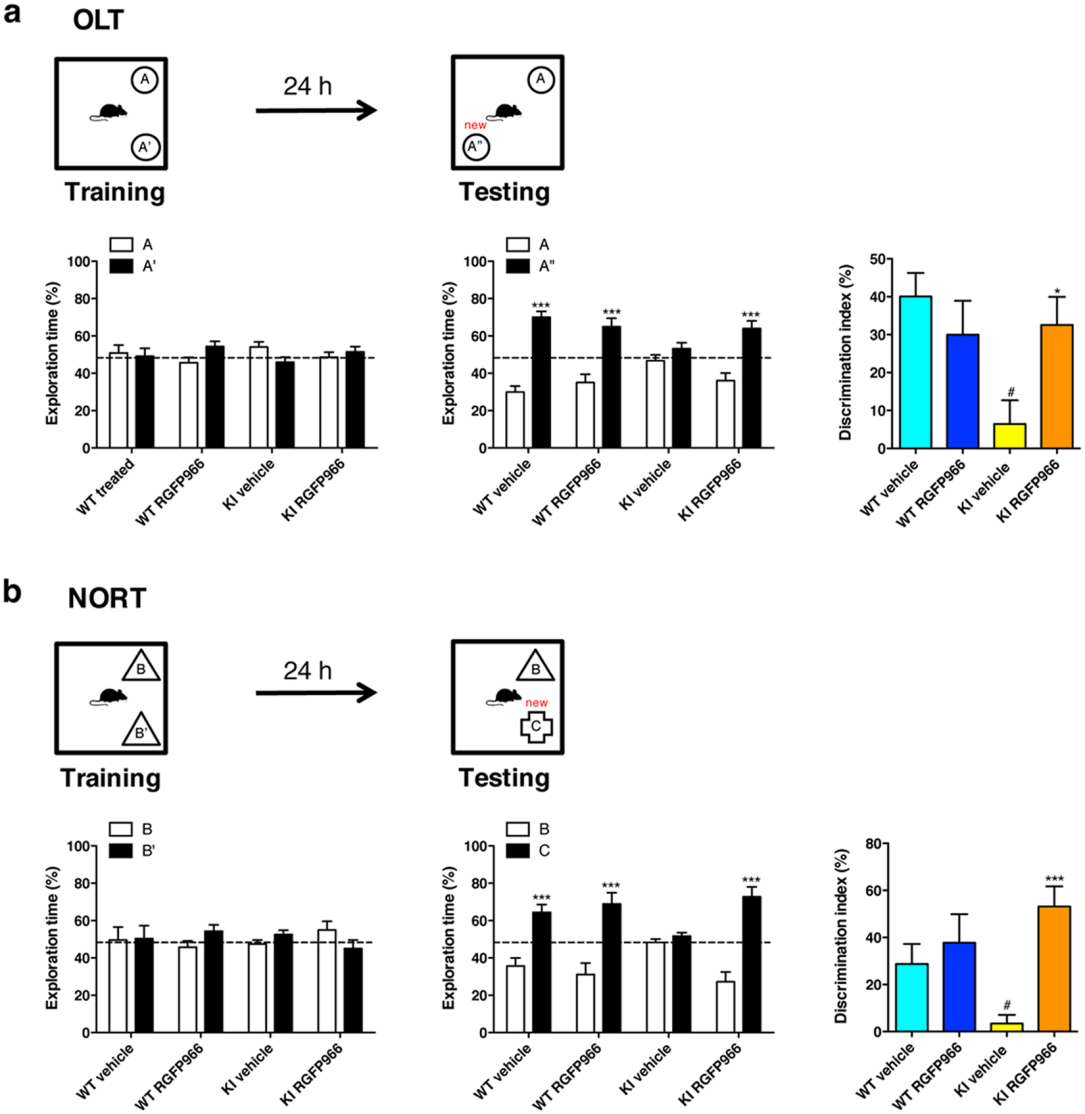
RGFP966 treatment prevents impairment of long-term memory in HD mice. Behavior analysis in vehicle and RGFP966 chronically treated Hdh^Q7/Q7^ (WT) and Hdh^Q7/Q111^ (KI) mice. Performance in **(a)** spatial (OLT) and **(b)** recognition (NORT) memory tests. Exploration time during training and testing session (left and center panels). Dashed line shows chance level for exploration. ***p < 0.001 compared to the percentage of time exploring the familiar object by two-way ANOVA with Bonferroni *post-hoc* analysis. Discrimination index (right panel). *p < 0.05, ***p < 0.001 compared to vehicle-treated KI mice; ^#^ *p* < 0.05 compared to vehicle-treated WT mice by two-way ANOVA with Bonferroni *post-hoc* analysis. Data represent the mean ± SEM (n = 5–9 animals per group).

Performance in the object location task (OLT) and the novel object recognition task (NORT) were evaluated in vehicle and RGFP966-treated mice (Fig. 2). In both tests, mice were first subjected to a training session in the presence of two similar objects. During training (Fig. 2a and b), the animals showed similar time exploring each object, indicating no detectable preference for object or location. When long-term memory was assessed 24 h after training (Fig. 2a and b), wild type mice demonstrated a clear increase in time exploring both the novel location and object regardless whether the animals were treated with RFGP966 or vehicle alone. In contrast, vehicle-treated KI mice demonstrated a similar time exploring familiar and novel objects, with minimal preference for either spatial or object novelty. This significant loss in recognition of object and spatial changes is consistent with previous studies of these KI mice^39^. Interestingly, treatment of KI mice with RGFP966 prevented the loss of spatial and recognition memories, as shown by increasing time exploring the novel versus the familiar object and a significantly higher discrimination index compared to vehicle-treated KI animals (Fig. 2a and b). These findings indicate that RGFP966 treatment prevents impairment in spatial and recognition long-term memory in HD mice.

### RGFP966 normalizes hippocampal expression of memory-dependent genes

**Figure 3:**
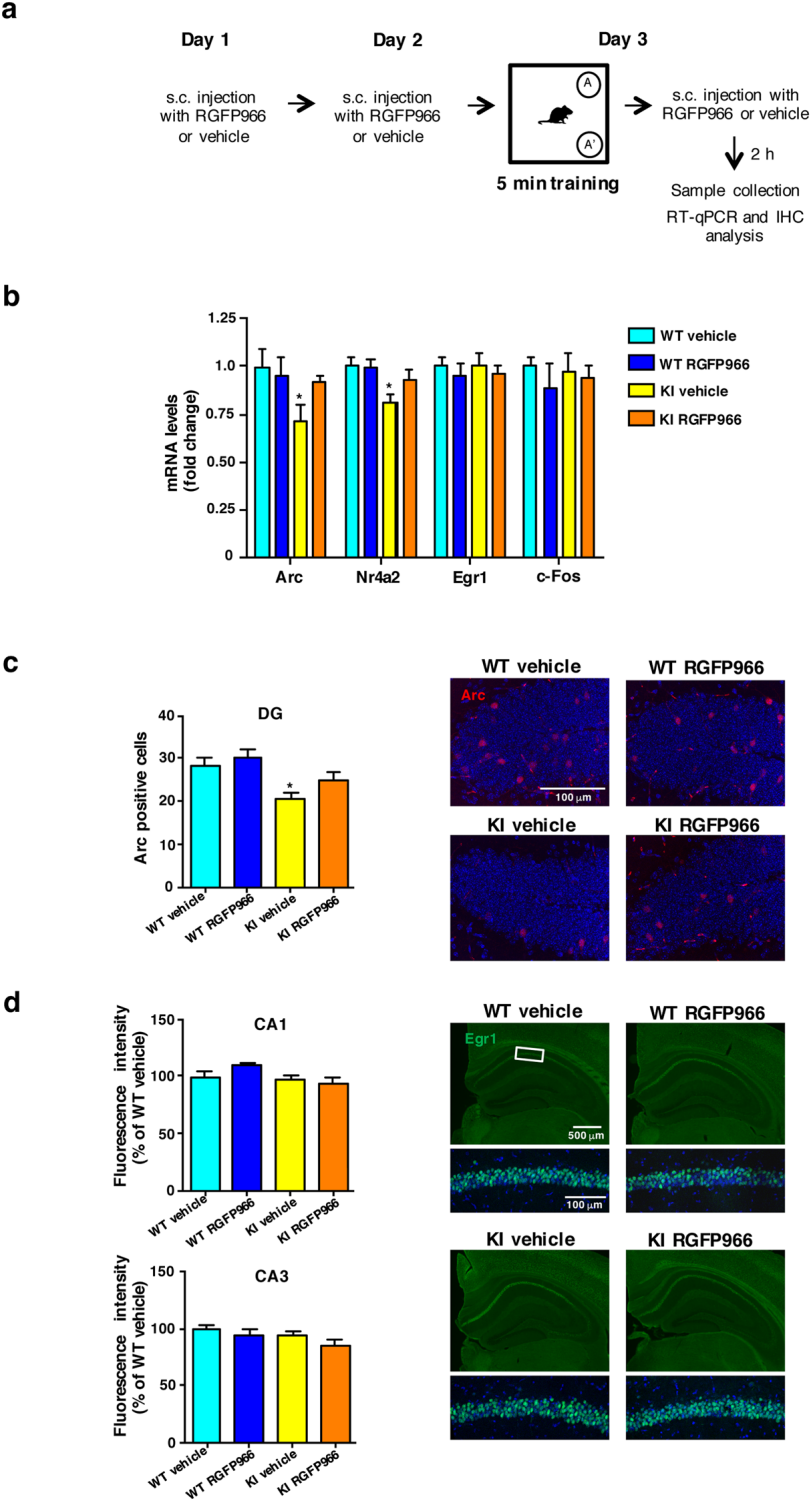
RGFP966 treatment restores gene expression in the hippocampus of HD mice. **(a)** Schematic time line of acute treatment. (**b)** Quantitative RT-PCR analysis of memory-related genes in the hippocampus of vehicle and RGFP966 acutely treated Hdh^Q7/Q7^ (WT) and Hdh^Q7/Q111^ (KI) mice. Histogram represents relative mRNA abundance of *Arc, Nr4a2, Egr1* and *c-Fos*. Levels of mRNA were normalized to 18S and Actinβ. *p < 0.05 compared to vehicle-treated WT mice by two-way ANOVA with Bonferroni *post-hoc* analysis. **(c)** Representative images (high magnification) showing Arc immunostaining in the hippocampal dentate gyrus of acutely treated Hdh^Q7/Q7^ (WT) and Hdh^Q7/Q111^ (KI) mice. Histogram shows quantification of the average number of Arc-positive neurons in the dentate gyrus. *p < 0.05 compared to vehicle-treated WT mice by two-way ANOVA with Bonferroni *post-hoc* analysis. **(d)** Representative images with magnification insets of the hippocampal CA1 region showing Egr1 immunostaining in acutely treated Hdh^Q7/Q7^ (WT) and Hdh^Q7/Q111^ (KI) mice. Histogram shows quantification of the average intensity of Egr1 immunoreactivity in the CA1 or the CA3 region. Data represent the mean ± SEM (n = 5–8 animals per group).

Recognition and spatial long-term memory deficits in KI mice have been associated with altered expression of activity-dependent, memory-related genes^7^. Inhibiting HDAC3 in hippocampus is predicted to elevate transcription of key genes for long-term memory formation in response to training^18^. To test this hypothesis, RT-qPCR was used to examine hippocampal expression of *Arc*, *Nr4a2, Egr1* and *c-Fos* after acute treatment of mice with RGFP966 or vehicle and in trained conditions (Fig. 3a). These genes are key neuronal activity-dependent early genes whose expression is required for long-term memory and synaptic plasticity^7, 44–46^ and some of them are known to be regulated by HDAC3 in a brain-region dependent manner^18, 19^. Arc and Nr4a2 transcript levels were significantly reduced in vehicle-treated KI mice compared to wild type animals while no changes were found in Egr1 and c-Fos expression. Interestingly, treatment of KI mice with RGFP966 led to Arc and Nr4a2 expression at levels indistinguishable from wild type mice (Fig. 3b). These results were confirmed by immunohistochemical analysis to detect Arc and Erg1 proteins. A significant decrease in the number of Arc-positive cells was detected in the dentate gyrus of vehicle-treated KI mice compared to wild type mice (Fig. 3c), and this decrease was blocked in RGFP966 treated KI mice. Arc-positive neurons were not evident in other hippocampal regions. In contrast, no significant differences between genotypes or treatment condition were found when Egr1 immunoreactivity was analyzed (Fig. 3d), in accordance with our data on mRNA gene expression (Fig. 3a). These findings suggest that HDAC3 inhibition affects the expression of certain memory-related genes.

**Figure 4:**
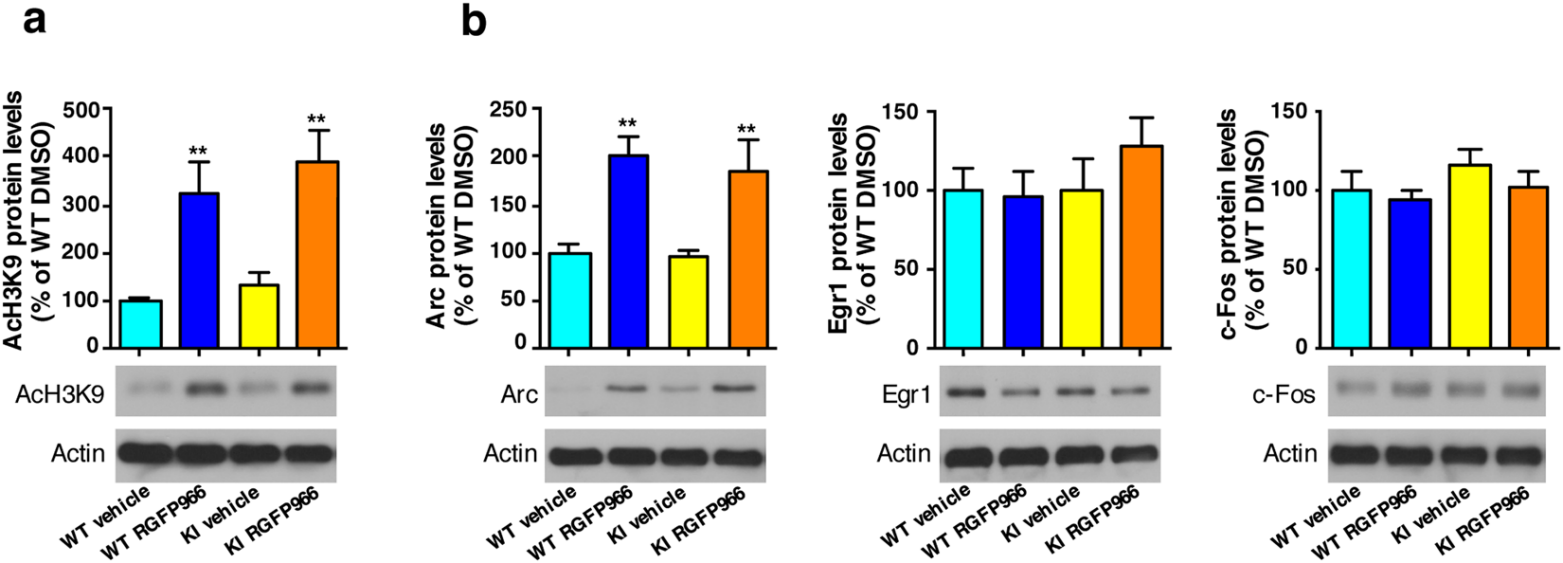
RGFP966 treatment induces Arc but not Egr1 or c-Fos protein levels in primary hippocampal cultures. **(a**) Representative immunoblots showing histone H3 acetylation levels at position lysine 9 (AcH3K9) with actin as loading control and **(b)** Arc, Egr1 and c-Fos protein levels normalized to actin levels in DMSO and RGFP966 treated primary hippocampal cultures obtained from Hdh^Q7/Q7^ (WT) and Hdh^Q7/Q111^ (KI) embryos. The blots in **(a** and **b)** are cropped; full-length images are provided in Supplementary Figure 3. **p < 0.01 compared to DMSO-treated cultures by two-way ANOVA with Bonferroni *post-hoc* analysis. Data represent the mean ± SEM (n = 4–5 cultures per group).

To better explore this selective-expression hypothesis, primary hippocampal cultures from wild type and KI embryos were incubated with vehicle or RGFP966 for 6 h followed by immunoblot analysis of H3K9 acetylation and of Arc, Egr1 and c-Fos protein levels. RGFP966 efficacy was indicated by a significant increase in H3K9 acetylation levels, compared to DMSO-treated controls, both in wild type and KI hippocampal neurons (Fig. 4a). Arc, Egr1 and c-Fos protein levels showed a differential response to the HDAC3 inhibitor. The same extracts from wild type and KI primary cultures revealed that RGFP966 treatment increased Arc protein levels without affecting levels of Egr1 or c-Fos regardless of genotype (Fig. 4b). Finally, to test whether HDAC1 and/or HDAC2 might also be affected at the inhibitor dose used, concentrations of RGFP966 below the IC_50_ of HDAC1/HDAC2^12, 19^ were tested in wild type and mutant huntingtin neuronal-like cell lines. Acetylation of H3 at lysine 9 (AcH3K9) and Arc protein levels were detected by immunoblot (Supplementary Fig. 1). Treatment with either 10 µM (Supplementary Fig. 1a) or 1 µM (Supplementary Fig. 1b) of RGFP966 led to drug-dependent increases of AcH3K9 in both wild type and mutant huntingtin striatal cells. In accordance with HDAC3 inhibition, protein levels of Arc were found significantly increased in RGFP966-treated versus vehicle-treated striatal cells. These data demonstrate that doses of RGFP966 with low activity for HDAC1 and HDAC2 elicit similar results than those at higher doses, suggesting a major contribution of HDAC3 inhibition on improvements in HD mice disease phenotypes.

Consistently, the results of transcript levels, immunohistochemistry and immunoblot analysis all suggest that RGFP966 ameliorates hippocampal-dependent memory deficits in KI mice by preventing detrimental reductions in expression levels in a specific subset of memory-related genes.

### RGFP966 prevents deficits in corticostriatal-dependent motor learning

**Figure 5:**
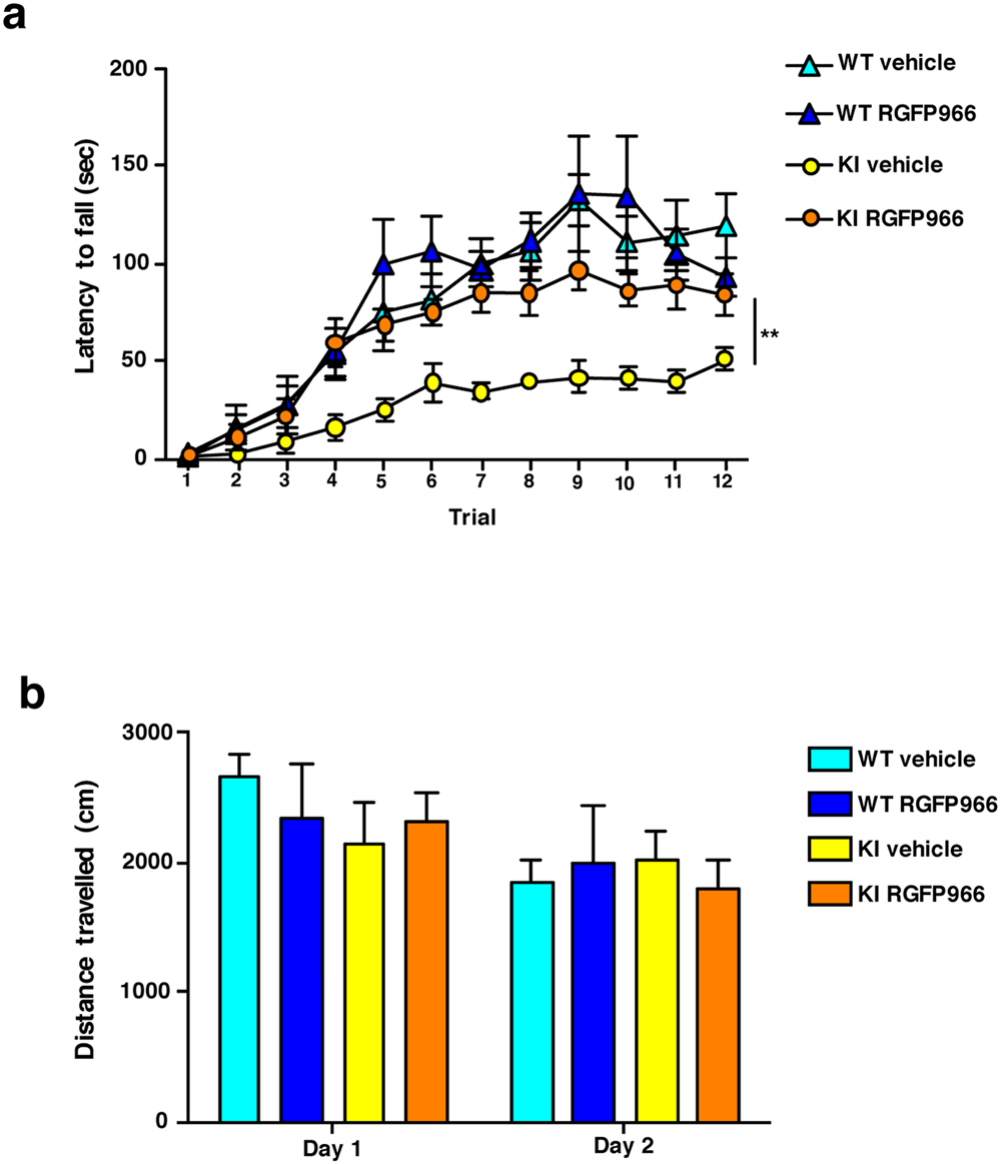
RGFP966 treatment prevents impairment of motor learning in HD mice. Behavior analysis in vehicle and RGFP966 chronically treated Hdh^Q7/Q7^ (WT) and Hdh^Q7/Q111^ (KI) mice. **(a)** Latency to fall in the accelerating rotarod task. **p < 0.01 compared to vehicle-treated WT mice by two-way ANOVA with repeated measurements. **(b)** Spontaneous locomotor activity measured by total distance traveled during two consecutive days (15 min/day). Data represent the mean ± SEM (n = 5–9 animals per group).

Impaired acquisition of new skills involving corticostriatal circuits has been reported in HD mice^40, 47, 48^ and HD patients^49–51^. Therefore, we next determined whether RGFP966 treatment of KI mice could also prevent the learning of new motor skills as an important example of corticostriatal-dependent behavior. Performance on an accelerating rotarod was evaluated in vehicle-treated and RGFP966-treated wild type and KI mice at 6 months of age (Fig. 5a). Both groups of wild type mice exhibited good motor learning ability over the trials. In contrast, vehicle-treated KI mice performed significantly worse with shorter latency to fall. Importantly, RGFP966-treated KI mice were able to learn to stay on the rotarod as well as vehicle- or RGFP966-treated wild type mice, indicating prevention of motor learning deficits upon treatment with the HDAC3-selective inhibitor. Since poor performance of vehicle-treated KI animals in the accelerating rotarod could be due to motor deficits, spontaneous locomotor activity in the open field was also evaluated. No significant differences in the distance traveled were found between genotypes and treatments (Fig. 5b). These results, along with previous data showing no deficits of KI mice in the fixed rotarod at six months of age^40^, indicate that the results obtained with the accelerating rotarod task were due to motor learning and not motor coordination deficits. Overall, these findings demonstrate that impaired ability to learn new corticostriatal-dependent motor skills can be prevented in KI mice by early treatment with RGFP966.

### RGFP966 suppresses striatal CAG repeat expansions

**Figure 6:**
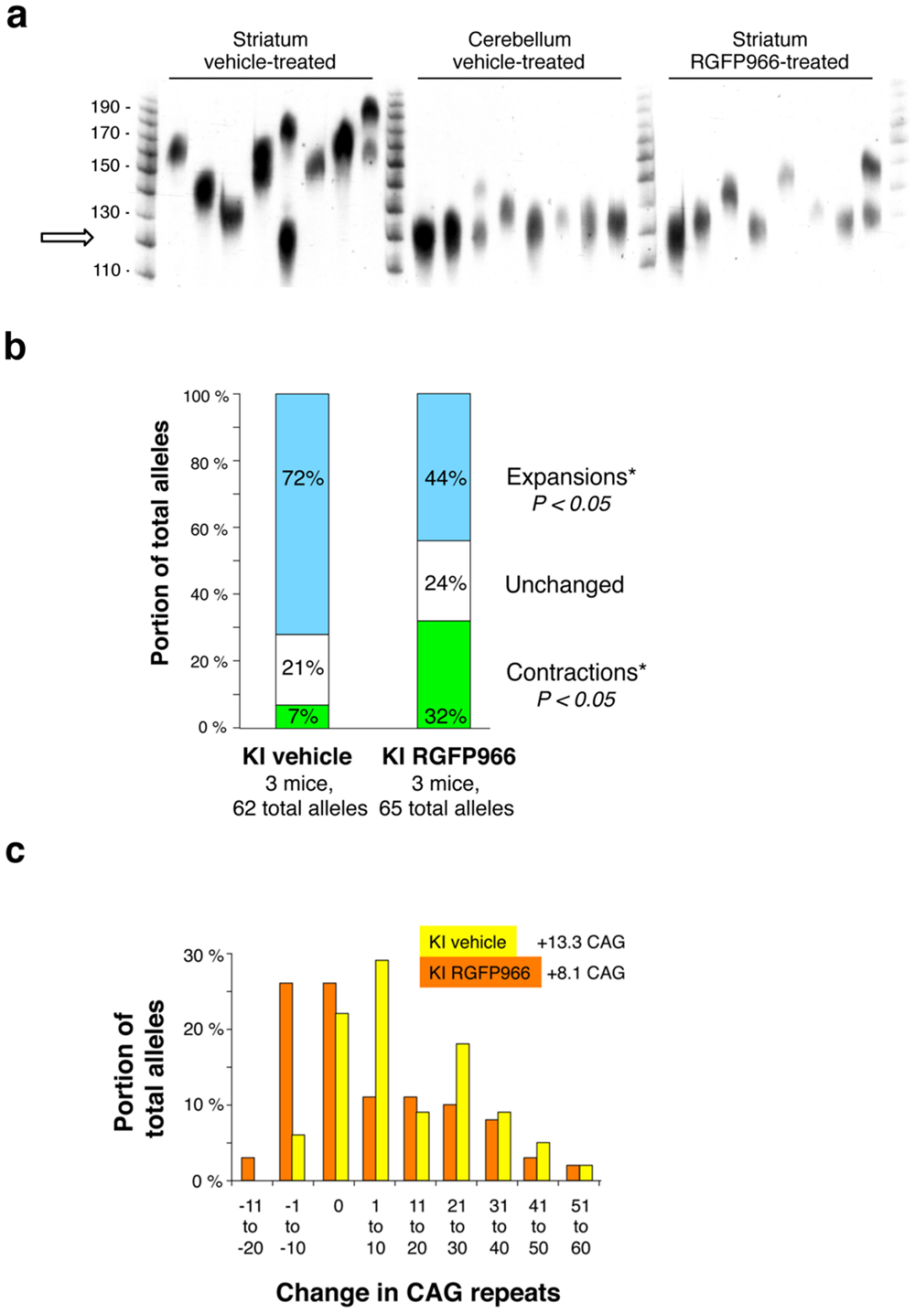
RGFP966 suppresses striatal CAG repeat expansions. **(a)** Representative display of small-pool PCR of somatic repeat lengths from single Hdh^Q7/Q111^ (KI) animals, either chronically treated with vehicle or with RGFP966. Size markers correspond to CAG repeat number. The examples shown represent the positive signals from one analysis. Arrow indicates size of initial CAG tract. This image has been cropped; the original is provided in Supplementary Figure 4. (**b**) Summary of genetic changes. For each group, n = 3 mice, 62–65 independent events. Starting tract sizes, estimated from the most common allele size in cerebellum and stated as repeat units, were 110–119, 120–129 and 120–129 for vehicle-treated mice; and 120–129, 120–129 and 130–139 for RGFP966-treated mice. *p < 0.05 by Student’s t-test. **(c)** Histogram of expansion sizes from the alleles tested in **b**. Allele sizes were binned into 10-repeat size groups, *e.g*. falling between +1 to +10 repeats compared to the starting allele size. Starting tract lengths were deduced from the most common allele size in the cerebellum of the same animals.

The molecular mechanisms underlying striatal pathology in HD are still being investigated. However, somatic *HTT* CAG expansions in striatum have been proposed as a mechanism that contributes to the pathogenic process^33–37^. We showed previously that HDAC3 stimulates CAG repeat expansions in cultured human astrocytes, and that a small molecule inhibitor of HDAC3 was as effective as RNAi knockdown at suppressing expansions^23, 24^. If the same holds true *in vivo*, one would predict lower levels of striatal expansions in the *HTT* CAG repeat following RGFP966 treatment. Somatic CAG repeat expansions were evaluated by small pool PCR (SP-PCR). This analysis provides quantitative assessment of expansion frequency as well as the size of individual expansions^32, 52^. Previous studies monitored expansions in the striatum, a tissue that shows substantial somatic instability and which is strongly affected in HD, and in cerebellum, where the CAG tract is relatively stable and mild pathology is found^25, 32^. Consistent with these previous studies, we saw substantial expansion activity in the striatum of vehicle-treated KI animals, whereas the CAG repeat was mostly stable in the cerebellum of the same mice (Fig. 6a). RGFP966 treatment partially stabilized the CAG repeat in striatum, showing fewer expansions as well as smaller changes in tract length (Fig. 6a). Quantitative data from three mice in each group, totaling 62–65 independent alleles, illustrated the beneficial effects of the HDAC3-selective inhibitor (Fig. 6b). Expansions predominated in the striatum of vehicle-treated KI animals (72%) compared to unchanged alleles (21%) and a few contractions (7%). These quantitative data are fully consistent with previous reports for HD mice^25, 32, 53^. In contrast, treatment with RGFP966 reduced the frequency of expansions by 28%, from 72% to 44% (Fig. 6b). Interestingly, this inhibition of expansions was compensated by a striking increase in the frequency of contractions from 7% in vehicle-treated KI mice to 32% in RGFP966-treated animals. Since the starting tract sizes were similar for all mice examined (legend to Fig. 6), differences in initial CAG repeat length were eliminated as a confounding variable.

The frequency data were corroborated by analysis of changes to CAG repeat lengths (Fig. 6c). Striatal changes in vehicle-treated KI animals spanned the range from −10 to +60 repeats with a weight-averaged length change of +13.3 repeats. RGFP966-treatment caused a leftward shift towards smaller size changes. The decrease was about 40% to a weight-average repeat change of +8.1 repeats. (Fig. 6c **)**. We conclude that RGFP966 treatment substantially suppressed striatal CAG repeat expansions in Hdh^Q7/Q111^ mice.

### RGFP966 partially rescues expression of striatal protein markers and reduces accumulation of mutant huntingtin oligomeric forms

**Figure 7:**
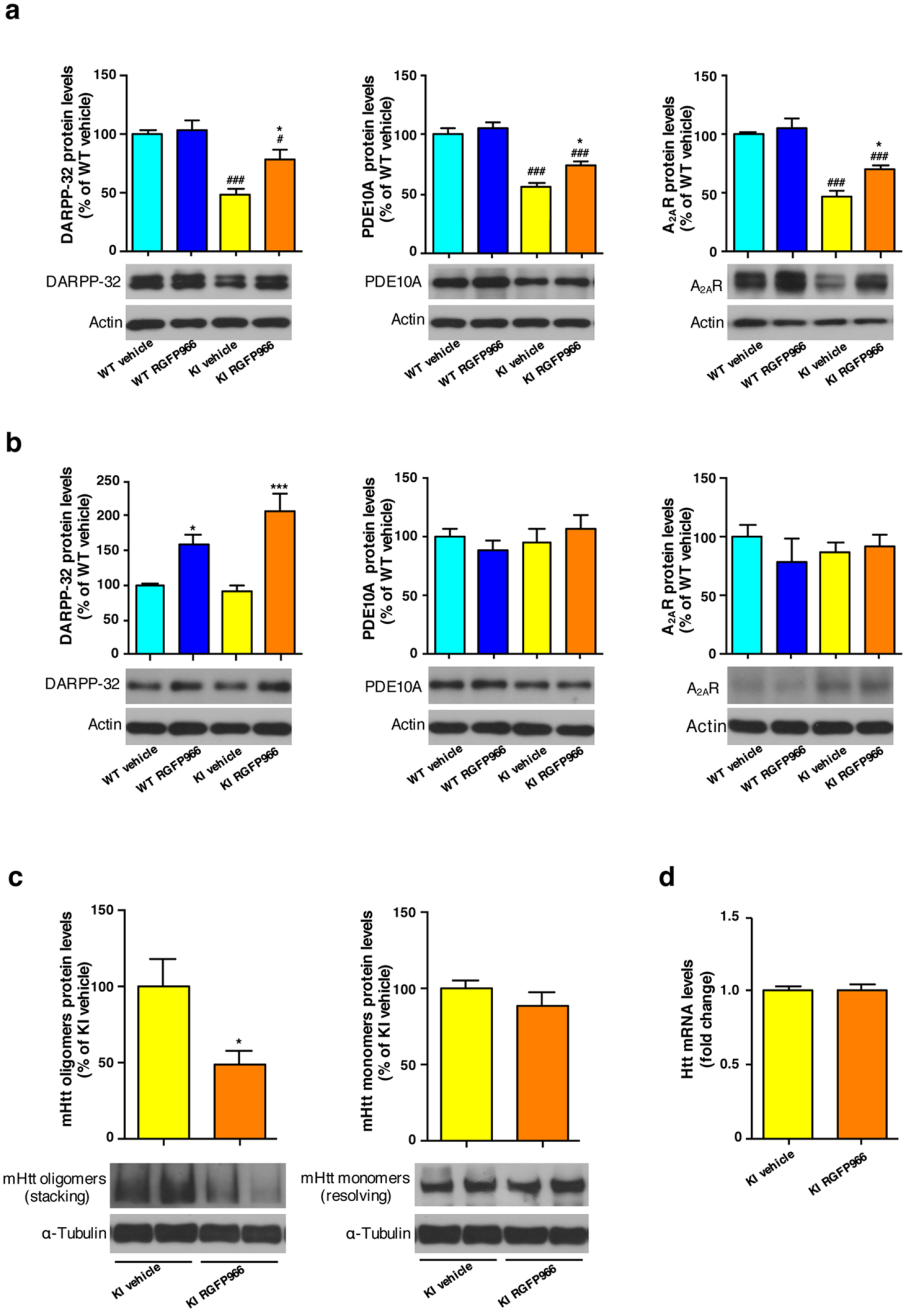
RGFP966 treatment ameliorates striatal pathology in HD mice. **(a)** Representative immunoblots showing striatal protein levels of DARPP-32, PDE10A and A_2A_R, with actin as loading control in vehicle and RGFP966 chronically treated Hdh^Q7/Q7^ (WT) and Hdh^Q7/Q111^ (KI) mice. *p < 0.05 compared to vehicle-treated KI mice; ^#^ *p* < 0.05; ^###^ *p* < 0.001 compared to vehicle treated WT mice by two-way ANOVA with Bonferroni *post-hoc* analysis. Data represent the mean ± SEM (n = 6–11 animals per group). **(b)** Representative immunoblots showing striatal protein levels of DARPP-32, PDE10A and A_2A_R with actin as loading control in DMSO and RGFP966 treated primary striatal cultures obtained from Hdh^Q7/Q7^ (WT) and Hdh^Q7/Q111^ (KI) embryos. *p < 0.05 and ***p < 0.001 compared to DMSO-treated cultures by two-way ANOVA with Bonferroni *post-hoc* analysis. Data represent the mean ± SEM (n = 4–5 cultures per group). **(c)** Representative immunoblots showing oligomeric forms of mutant huntingtin (stacking gel), monomeric mutant huntingtin (resolving gel) and α-tubulin as loading control in vehicle and RGFP966 chronically treated Hdh^Q7/Q111^ (KI) mice. Samples from two different mice are shown in adjoining lanes. All blots have been cropped; the original images are shown in Supplementary Figure 5. *p < 0.05 by Student’s t-test. Data represent the mean ± SEM (n = 6–10 animals per group). **(d)** Quantitative RT-PCR analysis of *Htt* mRNA in the striatum of vehicle and RGFP966 acutely treated Hdh^Q7/Q7^ (WT) and Hdh^Q7/Q111^ (KI) mice. Levels of mRNA were normalized to 18S and Actinβ. Data represent the mean ± SEM (n = 6–7 animals per group).

The integrity of striatal medium spiny neurons can be monitored by the level of protein biomarkers. Even at presymptomatic stages, reductions of phosphoprotein DARPP-32, phosphodiesterase PDE10A and adenosine receptor A_2A_R have been demonstrated as markers of dysfunctional striatal neurons^54–60^. To evaluate whether striatal improvements in RGFP966-treated KI mice were also associated with a recovery of these protein markers, levels of DARPP-32, PDE10A and A_2A_R were analyzed by immunoblot. As expected, levels of these striatal proteins were significantly reduced in vehicle-treated KI mice compared to wild type animals (Fig. 7a). Notably, RGFP966 treatment of KI mice partially prevented the reduction of these striatal markers, restoring them closer to normal levels. No significant effect of RGFP966 was observed in wild type mice. To elucidate whether the recovery of striatal expression of these protein markers was a direct effect of treatment with the HDAC3 inhibitor or a consequence of a general relief of striatal pathology, primary striatal cultures from wild type and KI embryos were incubated for 6 h with vehicle or RGFP966 and levels of DARPP-32, PDE10A and A_2A_R were subsequently analyzed by immunoblot (Fig. 7b). Though no significant differences in the expression of these striatal markers were found between DMSO-treated wild type and KI striatal cultures, RGFP966 treatment led to a significant increase in DARPP-32 levels in both genotypes (Fig. 7b). Inhibitor treatment was without any significant effect in PDE10A or A_2A_R protein levels. These results suggest a direct induction of DARPP32 expression as a result of RGFP966 treatment that contributes to ameliorating mHtt-induced striatal damage.

Oligomerization of mHtt in the striatum is another pathological hallmark of HD^61, 62^. Given the positive benefits of RGFP966 in striatal CAG expansions and protein biomarkers, we tested if RGFP966 treatment also reduces formation of mHtt oligomers. Detergent-soluble fractions of striatal extracts from KI mice were separated by SDS-PAGE. Mutant huntingtin oligomers were trapped in the stacking gel (Fig. 7c, left) whereas monomeric forms entered the resolving portion of the gel (Fig. 7c, right). Substantial accumulation of striatal mHtt oligomers was found in vehicle-treated KI mice, while treatment with RGFP966 prevented about one-half the accumulation of these oligomeric forms of mHtt (Fig. 7c). Monomeric mHtt levels were unchanged by inhibitor treatment. Next, we tested if RGFP966 alters the expression of the *Htt* gene at the transcriptional level. Quantitative RT-PCR analysis revealed no differences in *Htt* mRNA levels between acutely RGFP966-treated KI mice and vehicle-treated KI mice (Fig. 7d), suggesting that the reduction in mHtt oligomeric forms cannot be attributed to altered transcription of the *Htt* gene.

In sum, these observations demonstrate that the beneficial effects of RGFP966 were manifested both at the level of striatal protein markers and mHtt oligomerization.

## Discussion

This study shows that treating HD mice with an HDAC3-selective inhibitor provides multiple benefits. This conclusion is important because the success of HDAC inhibitors (HDACi) as a therapeutic approach for HD depends on the identification of key HDACs as targets. Selectively inhibiting one or a few key HDACs could provide therapeutic relief while avoiding toxicity issues that were encountered with broad-based HDACi. Selective HDAC3 inhibition has shown promise in improving HD motor impairments and neuropathology^8–12^. However, potential benefits of HDAC3 inhibition to cognitive function in HD have been largely unexplored. The current study demonstrates that early intervention with the HDAC3-selective inhibitor RGFP966 prevented deficits in hippocampal-dependent long-term memory likely by normalization of the expression in a specific subset of memory-related genes. In addition, RGFP966 suppressed striatal mHtt-induced protein toxicity by reducing both the frequency of somatic CAG expansions and the average change in repeat length which was associated with improvement of striatal pathology and prevention of motor skill learning deficits. While we cannot rigorously rule out some activity on HDAC1 or HDAC2 at the RGFP966 dose used here, these findings represent novel outcomes of inhibiting HDAC3 in HD mice and complement the work of Thomas and colleagues on motor function and working memory^9–11^. Our results and those of Thomas and colleagues indicate that early targeting with an HDAC3-selective inhibitor significantly prevents or delays cognitive deficits, somatic CAG repeat expansions and motor dysfunction in HD mice.

Our results are in contrast to those of Moumne *et al*.^63^ who examined *Hdac3* ^+/−^ heterozygous R6/2 HD mice. They found no amelioration of physiological or behavioral phenotypes and no effect on molecular changes including dysregulated transcripts. Surprisingly, these heterozygous mice still express HDAC3 protein at 80% of the wild type levels. This modest effect on HDAC3 levels makes it possible that sufficient protein and enzymatic activity are present in the heterozygous animals to confer HDAC3 effects on symptoms and transcription.

### Gene expression and hippocampal-dependent memory

Our results demonstrate that hippocampal-dependent memory decline was prevented by RGFP966. To our knowledge this is the first study involving the HDAC3 isotype in HD long-term memory impairment. These findings are consistent with our previous data showing restoration of long-term memory by trichostatin A (TSA), a broad-based HDACi^7^, and they are in agreement with the role of HDAC3 as a negative regulator of long-term memory formation^18^. We found that treatment with RGFP966 improved memory performance and normalized hippocampal *Arc* and *Nr4a2* immediate early gene expression, likely by increasing histone acetylation. Accordingly, a significant increase in the hippocampal expression of *Nr4a2* associated with enhanced long-term memory has been reported in HDAC3 knockout mice or mice treated with broad-based or specific HDAC3 inhibitors^7, 18^. Similarly, treatment with RGFP966 to inhibit HDAC3 enhances the memory processes involved in extinction of drug-seeking behaviors by enhancing *Nr4a2* and *c-Fos* expression^19^. Additionally, *Arc* expression has been widely involved in maintenance of long-term potentiation and consolidation of spatial long-term memory^64, 65^. Interestingly, these genes are dependent on CREB:CBP-mediated gene expression and it has been demonstrated that HDAC3 can also repress CBP function by deacetylation^66, 67^. Therefore, we speculate that inhibition of HDAC3 by RGFP966 may enhance CBP-mediated transcription of *Arc* and *Nr4a2* necessary for memory formation, which may help ameliorate memory decline in HD mice. It is also possible that other non-histone substrates of HDAC3 are modulated by RGFP966 treatment, contributing to memory improvement in HD mice.

### Striatal benefits of RGFP966 treatment

In addition to improvements in hippocampal function, RGFP966 treatment also led to benefits in striatal function. To our knowledge, this is the first demonstration of an HDAC3 inhibitor preventing motor learning decline. This result is consistent with the opposing effects of CBP and HDAC3 in expression of striatal genes important for acquisition of new motor skills. For example, mice harboring either a mutation in the CREB binding domain of CBP or knockout for CREB exhibit impaired chromatin acetylation and motor learning deficits, suggesting that CBP-mediated transcription is important for the expression of genes involved in motor learning^68, 69^.

Our results also provide the first demonstration of an HDAC3-selective inhibitor suppressing striatal expansions. The parallels in suppression of CAG repeat expansions between cell culture^23^ and the striatum of mice (Fig. 6) are consistent with the idea that suppression occurs primarily through RGFP966 inhibition of HDAC3, not off-target inhibition of HDAC1 or HDAC2. The changes in CAG repeat instability are similar to effects that were seen in Msh3^+/−^ HdhQ^111^ mice^70^, suggesting a possible connection between HDAC3 and Msh3. Msh3 is a key subunit of the DNA mismatch repair protein complex MutSβ, which stimulates expansions^33, 70–72^. Msh3 is a limiting factor for expansions in myotonic dystrophy type 1 mice^73^ and for formation of MutSβ complex in human cells^74^. Msh3 has also been linked genetically to the expansion-driving activity of HDAC3 in immortalized human glial cells^24^. Thus RGFP966 inhibition of HDAC3 in KI mice phenocopies the Msh3^+/−^ effect on striatal expansions, consistent with the model that mammalian HDAC3 and MutSβ work in the same pathway to drive CAG repeat expansions^24^. However, this interpretation is speculative and more experiments will be necessary to define the precise contribution of HDAC3 inhibition in MutSβ activity.

Consistent with inhibition of somatic CAG expansions, we report that RGFP966 treatment also prevented accumulation of mHtt oligomeric forms in the striatum of KI mice. Protein inclusions comprised of N-terminal fragments of mHtt are a characteristic hallmark of the disease, though whether they play a protective role or a causative one in neurodegeneration is controversial. Smaller, soluble oligomeric forms of huntingtin formed early in the aggregation process are thought to confer toxic effects and contribute to early cell dysfunction^75, 76^. Therefore, a reduction in the level of mHtt toxic oligomers by HDAC3 inhibition is predicted to delay disease progression. Interestingly, a recent study found no changes in striatal levels of mhtt aggregates, measured by EM48 immunoreactivity, after chronic HDAC3 inhibition in an HD mouse model that overexpresses the N-terminal fragment of mhtt^12^. This suggests that HDAC3 inhibition could have a different effect on oligomeric versus aggregate/inclusion forms of mhtt.

Another concurrent benefit of RGFP966 in the striatum was the normalization of striatal biomarkers levels such as DARPP-32, PDE10A and A_2A_R. Indeed, HDAC3 has been related to the transcriptional mechanisms that regulate striatal DARPP-32 expression. Thus, treatment of primary striatal cultures with the HDAC1/HDAC3 inhibitor 4b increased the overall levels of H3 and H4 histone acetylation as well as mRNA expression and protein levels of DARPP-32^77^. Similarly, inhibition of HDAC3 in R6/2 transgenic mice with HDACi-136 completely restored the expression of DARPP-32 mRNA levels^10^. Consistent with these reports, we have found that treatment of primary striatal cultures with RGFP966 significantly increased DARPP-32 protein levels suggesting that HDAC3 inhibition could elicit striatal benefits by directly inducing expression of DARPP-32.

Overall these findings strengthen the link between somatic CAG expansions and HD progression. One speculative model is that fewer expansions and shorter changes to CAG repeat length would limit gains in the length of the glutamine tract of mHtt, reducing mHtt-induced toxicity and thereby leading to improvements in striatal function and recovery of motor learning dysfunction. This is consistent with the work of McMurray and colleagues. They showed that treatment of HD mice with XJB-5-131, a bifunctional antioxidant, led to enhanced neuronal survival, suppression of motor decline and weight loss, improved mitochondrial function and inhibition of somatic CAG repeat expansions^37, 78^.

In summary, our study demonstrates that early targeting with an HDAC3-selective inhibitor provides multiple benefits in HD mice by preventing hippocampal-memory impairments and by suppressing striatal degeneration. The finding that somatic CAG repeat expansions were also suppressed by the inhibitor may represent an interesting new therapeutic approach not only for HD but also for other trinucleotide repeat disorders.

## Methods

### Animals

Hdh^Q111^ knock-in mice, with targeted insertion of 109 CAG repeats that extends the glutamine segment in murine huntingtin to 111 residues, were maintained on a C57BL/6 genetic background^79^. Female Hdh^Q7/Q7^ mice were crossed with male Hdh^Q7/Q111^ mice to generate age-matched Hdh^Q7/Q7^ wild type (WT) and Hdh^Q7/Q111^ knock-in (KI) littermates, determined by PCR analysis. Only males were analyzed. Mice were housed with access to food and water *ad libitum* in a colony room kept at 19–22 °C and 40–60% humidity, under a 12:12 h light/dark cycle. All procedures involving animals were performed in compliance with the National Institutes of Health Guide for the Care and Use of Laboratory Animals, and approved by the local animal care committee of the Universitat de Barcelona (99/01) and Generalitat de Catalunya (99/1094), in accordance with the European (2010/63/EU) and Spanish (RD53/2013) regulations for the care and use of laboratory animals.

### RGFP966 mice treatment

The HDAC3 inhibitor RGFP966 was generously provided by BioMarin Pharmaceutical Inc. (San Rafael, California, USA). For evaluation of behavior, biochemical parameters and CAG repeat expansions, WT and KI mice were subcutaneously injected with 50 mg/kg of RGFP966 or vehicle (70% polyethylene glycol 200; 30% acetate buffer) three times per week from 3 to 6.5 months (chronic treatment). Mice were sacrificed by cervical dislocation 1 hour after final injection and brains were removed for immunoblot and CAG repeat analysis. For analysis of activity-dependent genes, 7-month-old WT and KI mice were subcutaneously injected daily with 25 mg/kg of RGFP966 or vehicle for 3 days (acute treatment). On day 3, mice received 5 min training in an environment with two identical objects to induce expression of activity-dependent memory genes. Training was immediately followed by an injection of RGFP966. Mice were sacrificed 2 hours later and their brains removed for gene expression and histological analysis.

### Behavioral assessment

Chronically treated animals were evaluated for behavior at 6 months of age. Motor learning was assessed by the accelerating rotarod task while spatial and recognition long-term memory was assessed by the object location task (OLT) and the novel object recognition task (NORT), respectively. Locomotor activity was evaluated by the open field test^39, 40^.

#### Accelerating rotarod training procedure

Animals were placed on a motorized rod (30 mm diameter) and the rotation speed was gradually increased from 4 to 40 rpm over the course of 5 minutes. Time latency was recorded when the animal was unable to keep up with the increasing speed and fell. Accelerating rotarod training procedure training/testing was performed four times per day for three consecutive days. Different trials during the same day were separated by 1 h. The apparatus was rigorously cleaned between animal trials in order to avoid odors.

#### Open field

The device consisted of a white open-top arena with quadrangular form (45 × 45 cm). The light intensity was 40 lux throughout the arena, and the room temperature was kept at 19–22 °C and 40–60% humidity. Mice were placed into the arena during two consecutive days (15 min/day) and spontaneous locomotor activity was measured as total distance traveled. The arena was rigorously cleaned between animals in order to avoid odors. Animals were tracked and recorded with SMART Junior Software.

#### OLT/NORT

Exploration took place in an open-top arena with quadrangular form (45 × 45 cm). The light intensity was 40 lux throughout the arena, and the room temperature was kept at 19–22 °C and 40–60% humidity. Mice were first habituated to the arena in the absence of objects (2 days, 15 min/day). On the third day during the acquisition phase mice were allowed to explore 2 duplicate objects (A and A′ or B and B′), which were placed in two adjacent corners of the arena, for 10 min, after which they were returned to their home cage. During the 24 h retention test, mice were placed in the experimental apparatus for 10 min. For OLT, one copy of the familiar object (A) was placed in the same location as during the training trial, and one copy of the familiar object (A″) was placed in the corner diagonally opposite. For NORT, one copy of the familiar object (B) and a new object (C) were placed in the same location as during the training trial. The arena was rigorously cleaned between animal trials in order to avoid odors. Animals were tracked and recorded with SMART Junior software. Exploration times were recorded and used to calculate the Discrimination index = (time exploring novel or relocated object-time exploring familiar object)/(total time exploring both objects) × 100. Discrimination indices of 0 indicate equal exploration of both objects.

### Immunoblot analysis

Brains from chronically treated mice were quickly removed, dissected, frozen in dry ice and stored at −80 °C until use. Brain tissue was homogenized in cold lysis buffer containing 20 mM Tris base (pH 8.0), 150 mM NaCl, 50 mM NaF, 1% NP-40, 10% glycerol and supplemented with 1 mM sodium orthovanadate and protease inhibitor cocktail (Sigma-Aldrich). Samples were centrifuged at 16,000 g for 20 min and the supernatants collected. For the analysis of cellular extracts, cells were collected in cold lysis buffer containing 50 mM Tris base (pH 7.4), 150 mM NaCl, 2 mM EDTA, 0.1 mM phenylmethylsulfonyl fluoride, 1% NP-40 and supplemented with 1 mM sodium orthovanadate and protease inhibitor cocktail. Samples were centrifuged at 10,000 g for 10 min, and the supernatants collected. Following determination of the protein contents by Detergent-Compatible Protein Assay (Bio-Rad, Hercules, CA, USA), protein extracts (20–60 µg) were mixed with 5 × SDS sample buffer, boiled for 5 min, resolved on 6–10% SDS–PAGE and transferred to nitrocellulose membranes (Whatman Schleicher & Schuell, Keene, NH, USA). After incubation (30 min) in blocking buffer containing 10% non-fat powdered milk in Tris buffered saline-Tween (TBS-T) (50 mM Tris–HCl, 150 mM NaCl, pH 7.4, 0.05% Tween 20), membranes were blotted overnight at 4 °C with primary antibodies. Antibodies used for immunoblot analysis were: Acetyl-histone H3 (Lys9) (1:1000, Cell Signaling Technology), Histone H3 (1:1000, Cell Signaling Technology), HDAC3 (1:1000; Abcam), Arc (1:500; Santa Cruz Biotechnology), Egr1 (1:1000; Cell Signaling Technology), c-Fos (1:1000, Santa Cruz Biotechnology), DARPP-32 (1:1000; BD Bioscience), PDE10A (1:1000; Abcam), A_2A_R (1:500; Santa Cruz Biotechnology), 1C2 (1:1000, Millipore), α-Tubulin (1:50,000; Sigma-Aldrich) and Actin (1:20,000; MP Biochemicals). The membranes were then rinsed three times with TBS-T and incubated with horseradish peroxidase-conjugated secondary antibody for 1 h at room temperature. After washing for 30 min with TBS-T, the membranes were developed using the enhanced chemilluminescence ECL kit (Santa Cruz Biotechnology). The Gel-Pro densitometry program (Gel-Pro Analyzer for Windows, version 4.0.00.001) was used to quantify the different immunoreactive bands relative to the intensity of the α-tubulin, actin or histone H3 band in the same membranes within a linear range of detection for the ECL reagent.

### Quantitative RT-PCR

Brains from acutely-treated mice were quickly removed, dissected, frozen in dry ice and stored at −80 °C until use. Total RNA was isolated using the total RNA isolation Nucleospin RNA II Kit (Macherey-Nagel). Purified RNA (500 ng) was reverse transcribed using the PrimeScript RT Reagent Kit (Perfect Real Time, Takara Biotechnology Inc.). The cDNA synthesis was performed at 37 °C for 15 min and a final step at 85 °C for 5 s in a final volume of 20 μl according to the manufacturer’s instructions. The cDNA was then analyzed by quantitative RT-PCR using the following PrimeTime qPCR Assays (Integrated DNA Technologies, Inc.): Arc (Mm.PT.56a.16160059), Nr4a2 (Mm.PT.58.16021564), Egr-1 (Mm.PT.58.29064929); c-Fos (Mm.PT.58.29977214), Htt (Mm.PT.58.12088552, 18S (Hs.PT.39a.22214856.g) and Actinβ (Mm.PT.39a.22214843.g). Quantitative PCR was performed in 12 μl of final volume on 96-well plates using the Premix Ex Taq (Probe qPCR) (TAKARA BIOTECHNOLOGY (Dalian) Co., LTD). Reactions included Segment 1:1 cycle of 30 s at 95 °C and Segment 2: 40 cycles of 5 s at 95 °C and 20 s at 60 °C. All quantitative PCR assays were performed in duplicate. To provide negative controls and exclude contamination by genomic DNA, the PrimeScript RTEnzyme was omitted in the cDNA synthesis step. The quantitative PCR data were quantified using the comparative quantitation analysis program of MxProTM quantitative PCR analysis software version 3.0 (Stratagene) using 18S and Actinβ gene expression as housekeeping genes.

### Immunohistochemical analysis

Brains from acutely treated mice were fixed by immersion in paraformaldehyde (Sigma-Aldrich) 4% in PBS, cryoprotected in PBS/sucrose 30% with 0.02% sodium azide and frozen in methyl–butane (Sigma-Aldrich). Coronal sections (30 µm) of the brain were obtained using a cryostat (Microm) and kept in PBS. Free-floating sections were rinsed three times in PBS and permeabilized and blocked in PBS containing 0.3% Triton X-100 and 3% normal goat serum (Pierce Biotechnology, Rockford, IL) for 15 min at room temperature. Sections were then washed in PBS and incubated overnight at 4 °C with mouse anti-Arc (1:200, Santa Cruz Biotechnology) or rabbit anti-Egr1 (1:200, Cell Signaling Technology). After primary antibody incubation, slices were washed three times and then incubated 2 h shaking at room temperature with secondary antibodies: anti-rabbit Alexa Fluor 488 (1:100, Jackson ImmunoResearch, West Grove, PA, USA) or anti-mouse Cy3 (1:100, Jackson ImmunoResearch, West Grove, PA, USA). Nuclei were stained with Hoechst 33258 (1:10,000, Molecular Probes, Life Technologies) for 10 min. As negative controls, some sections were processed as described in the absence of primary antibody and no signal was detected. Images were acquired with a Leica SP5 laser scanning confocal microscope (Leica). For each mouse, at least four slices of 30 μm-containing hippocampal tissue were analyzed. Arc immunoreactive-positive positive cells in the dentate gyrus were counted and Egr1 immunoreactivity in the CA1 or CA3 area of the hippocampus was quantified by analysis of integrated optical density with ImageJ software, as previously described^80^.

### Primary neuronal cultures

Dissociated hippocampal and striatal cultures prepared from E18.5 WT and KI embryos were plated at a density of 400,000 neurons onto 60 mm culture dishes precoated with 0.1 mg/ml poly-d-lysine (Sigma Chemical Co., St. Louis, MO). Neurons were cultured in Neurobasal medium (Gibco-BRL, Renfrewshire, Scotland, UK), supplemented with B27 (Gibco-BRL) and Glutamax™ (Gibco-BRL) and were maintained at 37 °C in a humidified atmosphere containing 5% CO2. At 10–14 DIV, neurons were treated with vehicle (DMSO) or RGFP966 (10 μM) for 6 h. Additionally, primary hippocampal cultures were treated with 50 mM KCl for the last 2 hours in order to induce neuronal stimulation. Subsequently, cells were harvested for immunoblot analysis.

### Immortalized cell culture

Conditionally immortalized wild-type STHdh^Q7/Q7^ and mutant STHdh^Q111/Q111^ striatal neuronal progenitor cell lines expressing endogenous levels of normal and mutant huntingtin with 7 and 111 glutamines, respectively, have been described previously^81^. Striatal cells were grown at 33 °C in Dulbecco’s modified Eagle’s medium (DMEM, Sigma-Aldrich; St. Louis, MO, USA), supplemented with 10% fetal bovine serum, 1% penicillin–streptomycin, 2 mM l-glutamine, 1 mM sodium pyruvate and 400 μg/ml G418 (Geneticin; GIBCO-BRL, Life technologies; Gaithersburg, MD, USA). Cells were treated with vehicle (DMSO) or RGFP966 (10 µM or 1 µM) for 6 h prior to harvesting for immunoblot analysis.

### Striatal CAG repeat instability

CAG repeat expansions were detected by small-pool PCR (SP-PCR) using primers specific for the *HTT* allele^70^. Genomic DNA was prepared from mouse tissue using Macherey-Nagel NucleoSpin Tissue Kit following the manufacturer’s protocol. One μg of genomic DNA was digested with 20 U EcoRV-HF at 37 °C for 1.5 hours then heat inactivated at 65 °C for 10 minutes. Digested DNA was serially diluted in TE buffer (pH 8) containing 0.1 μM carrier primer (CAG1_HdhQ111_F) to give a final concentration of 20 pg/μl. CAG repeat sizes were determined by PCR using forward primer CAG1_HdhQ111_F (5′-atg aag gcc ttc gag tcc ctc aag tcc ttc-3′) and reverse primer HU3_HdhQ111_R (5′-ggc ggc tga gga agc tga gga-3′). The PCR reaction mixture contained 10 pg template DNA, 1X buffer (67 mM Tris-HCl pH 8.8, 16.6 mM NH_4_SO_4_, 2.0 mM MgCl_2_, 10 mM 2-mercaptoethanol) 10% DMSO, 0.17 mg/ml BSA, 0.2 mM each dATP, dCTP, dGTP and dTTP, 4 ng/μl primers and 0.5 U Taq polymerase (Fisher BioReagents). PCR reactions were performed in multiplex (20–30 reactions per sample) with at least four negative control reactions. PCR conditions were one cycle of 90 sec at 94 °C, 34 cycles of (30 sec at 94 °C, 30 sec at 65 °C and 90 sec at 72 °C) and one final cycle of 10 min at 72 °C. PCR products were denatured in formamide loading buffer (0.05% w/v Bromophenol Blue, 0.05% w/v Xylene cyanol, 96% v/v deionized formamide, 20 mM EDTA pH 8) at 95 °C for 8 minutes then resolved, alongside DIG labeled size markers, on a 6% denaturing sequencing gel in 1X TBE buffer at 60 W for ~4 hours. Fragments were transferred to positively charged nylon membrane by electroblotting at 30 V overnight at 4 °C and fixed by UV cross-linking. Southern blot hybridization was performed using a 5′ digoxigenin (DIG) labeled locked nucleic acid (LNA) probe GC*T G*CT GC*T G*CT GC*T GCT (Eurogentec, where C* indicates LNA cytosine and G* indicates LNA guanine). Detection was performed using DIG High Prime DNA labeling and Detection Starter Kit II (#11 585 614 910; Roche Applied Science, Mannheim, Germany) following the manufacturer’s protocol. Multiple exposures to X-ray film ensured detection of faint signals and separation of closely spaced bands. Starting tract size was deduced from the most common product length in the cerebellum of each animal.

### Statistical analysis

All statistical comparisons were performed using either two-way ANOVA or Student’s t-test, as indicated in figure legends. Values for n and *p* are given in the legend to each figure. Differences with *p* < 0.05 were considered significant.

## Electronic supplementary material

Supplementary material
