# Supplementary material for: A selective inhibitor of histone deacetylase 3 prevents cognitive deficits and suppresses striatal CAG repeat expansions in Huntington’s disease mice
Source: Sci Rep. 2017 Jul 20;7:6082. doi: 10.1038/s41598-017-05125-2 (PMC5519595; doi:10.1038/s41598-017-05125-2)
Supplement: Supplementary file 1 — Supplementary material [file 41598_2017_5125_MOESM1_ESM.pdf]

Supplementary Information

A selective inhibitor of histone deacetylase 3 prevents cognitive deficits and suppresses striatal CAG repeat expansions in Huntington´s disease mice

Nuria Suelves[1,2,3,4], Lucy Kirkham-McCarthy[5], Robert S. Lahue[5,6]* and Silvia Ginés[1,2,3,4]*

[1]Departament de Biomedicina, Facultat de Medicina, Universitat de Barcelona, Barcelona, Spain
[2]Institut d' Investigacions Biomèdiques August Pi i Sunyer (IDIBAPS), Barcelona, Spain
[3]Centro de Investigación Biomédica en Red sobre Enfermedades Neurodegenerativas (CIBERNED), Madrid, Spain
[4]Institut de Neurociències, Universitat de Barcelona, Barcelona, Spain
[5]Centre for Chromosome Biology, National University of Ireland Galway, Newcastle Road, Galway, Ireland
[6]NCBES Galway Neuroscience Centre, National University of Ireland Galway, Newcastle Road, Galway, Ireland

Correspondence should be addressed to R.S.L (bob.lahue@nuigalway.ie, +353-91495756) or S.G. (silviagines@ub.edu, + 34-934035284)

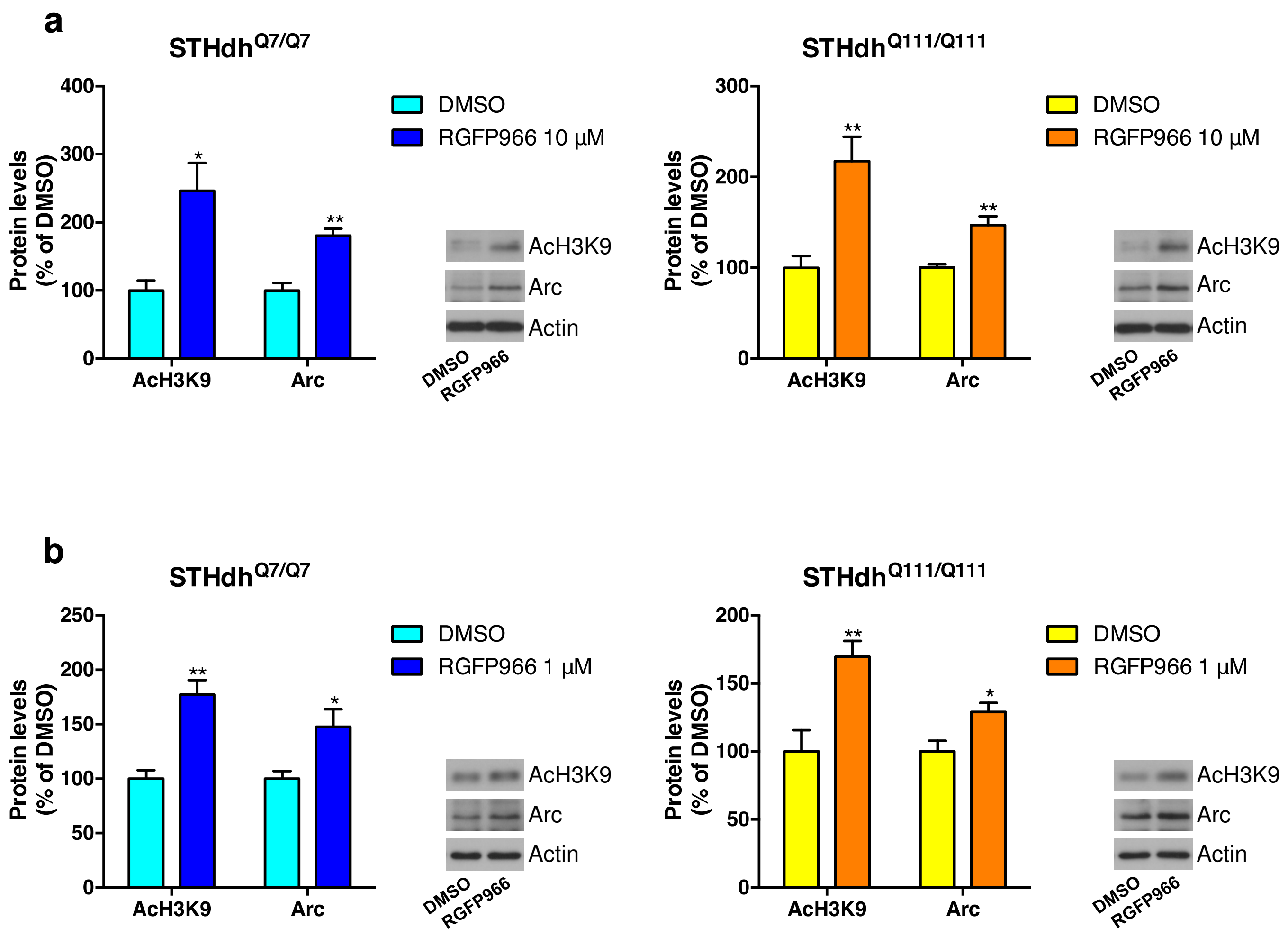

Supplementary Figure 1. RGFP966 treatment increases AcH3K9 and Arc protein levels in immortalized striatal cells. Representative immunoblots showing Arc and histone H3 acetylation levels at position lysine 9 (AcH3K9) with actin as loading control in STHdh$^{Q7/Q7}$ or STHdh$^{Q111/Q111}$ striatal cells treated with DMSO or RGFP966 at **(a)** 10 µM or **(b)** 1 µM. The blots in **(a)** and **(b)** are cropped; full-length images are provided in Supplementary Figure 6. *p<0.05 and **p<0.01 by Student's t-test. Data represent the mean ± SEM (n=4-5 cultures per group).

Figure 1c

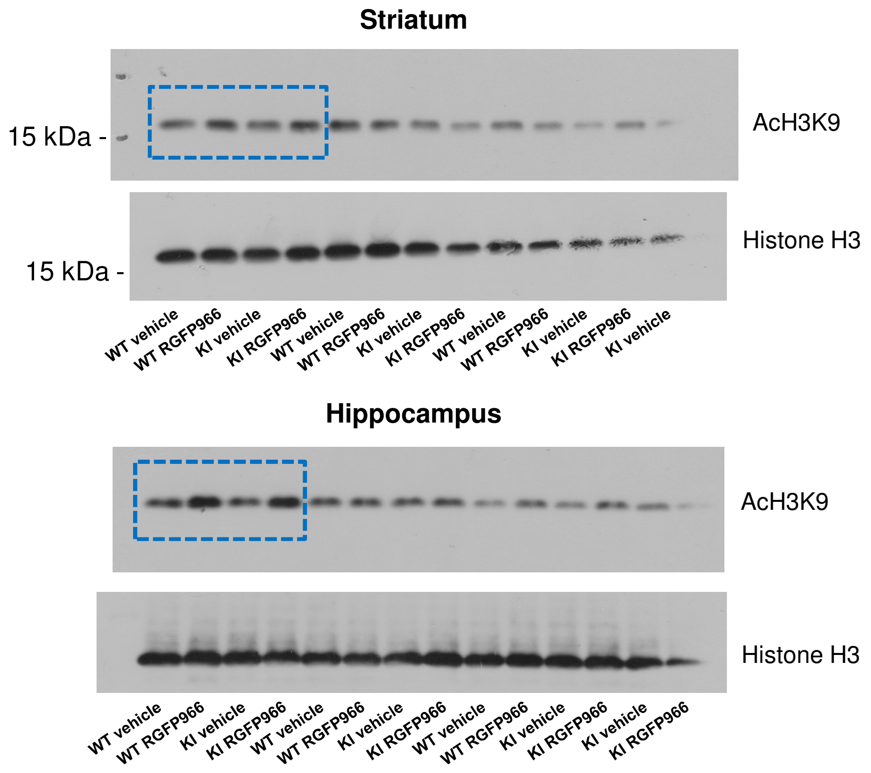

Figure 1d

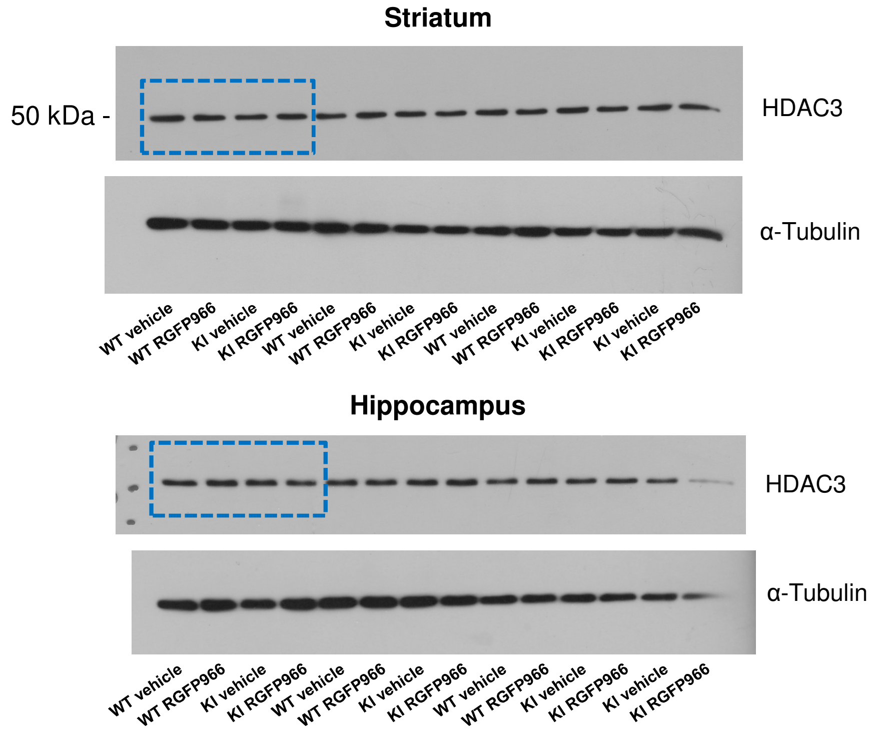

Supplementary Figure 2: original images of cropped immunoblots in Figure 1

Figure 4a

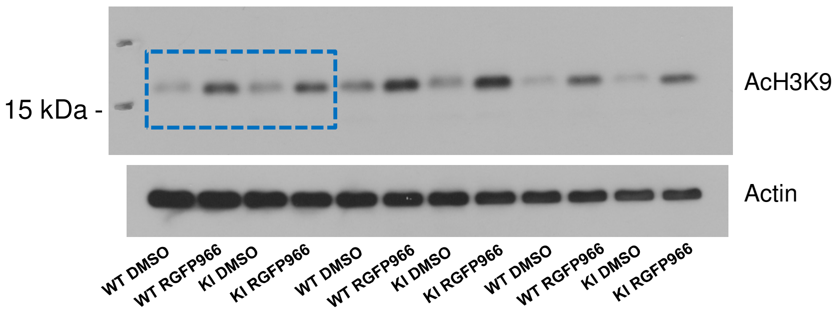

Figure 4b

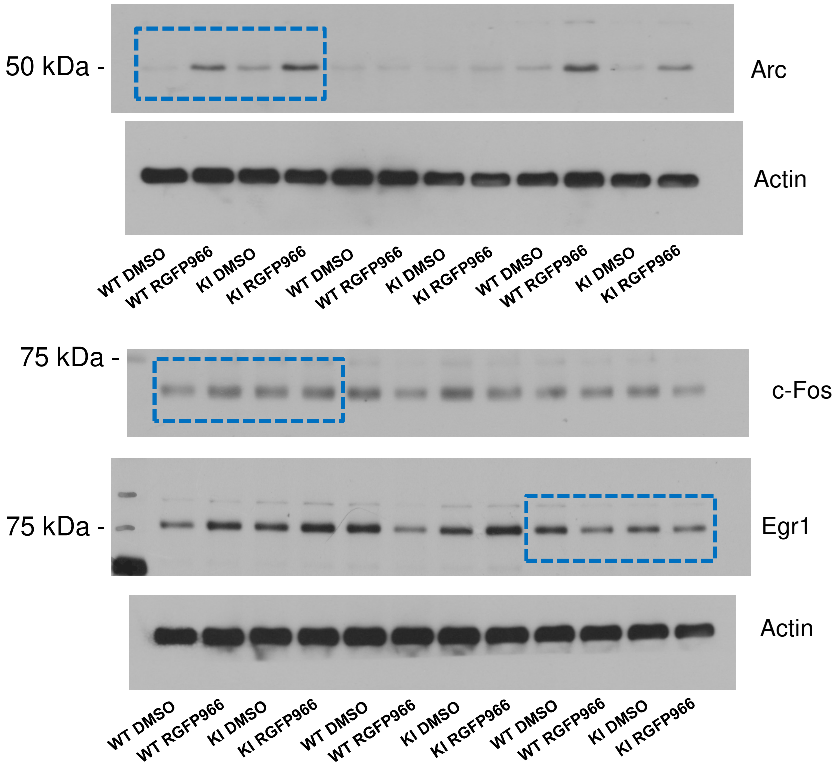

Supplementary Figure 3: original images of cropped immunoblots in Figure 4

Figure 6a

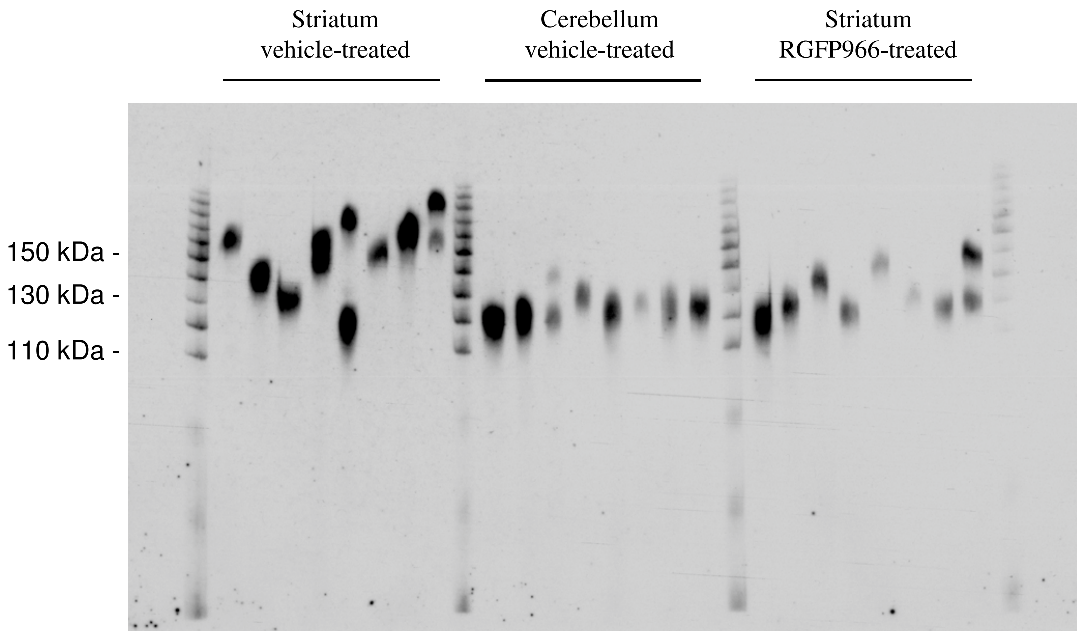

Supplementary Figure 4: original image of cropped immunoblot in Figure 6

Figure 7a

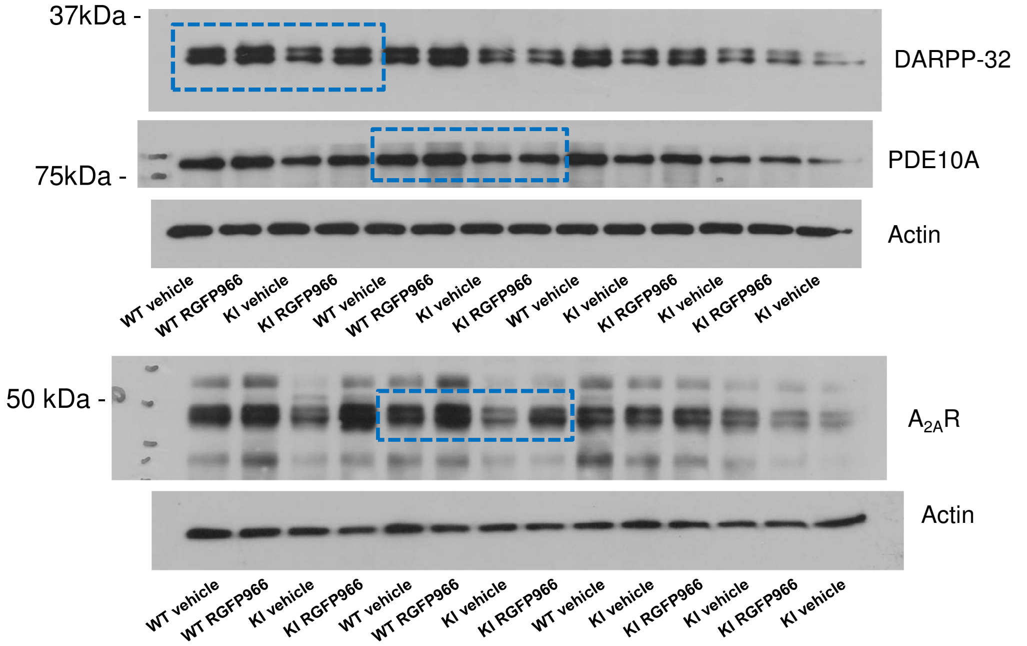

Figure 7b

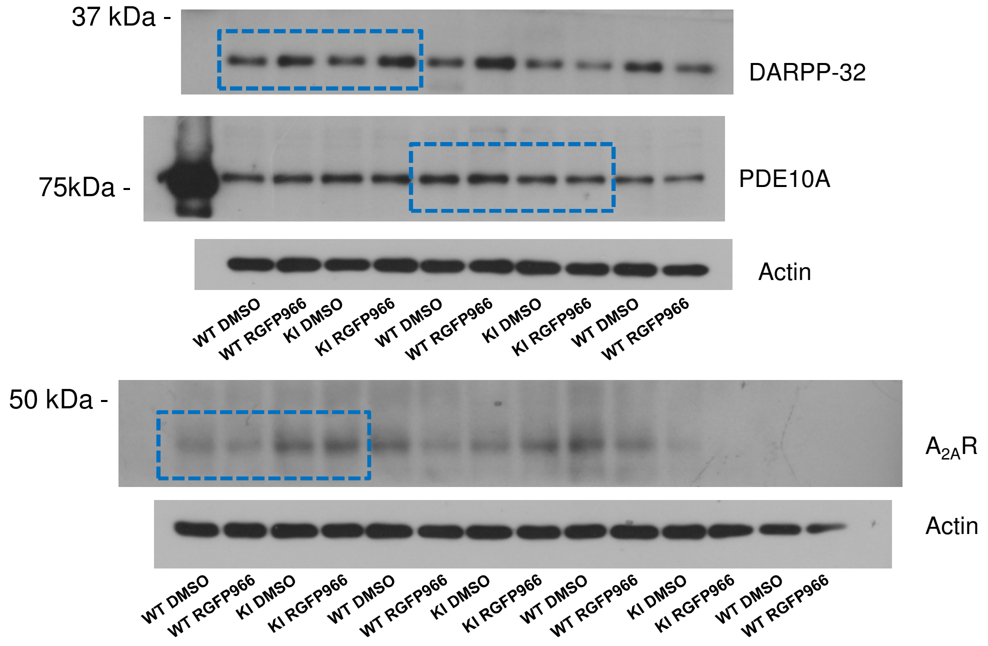

Figure 7c

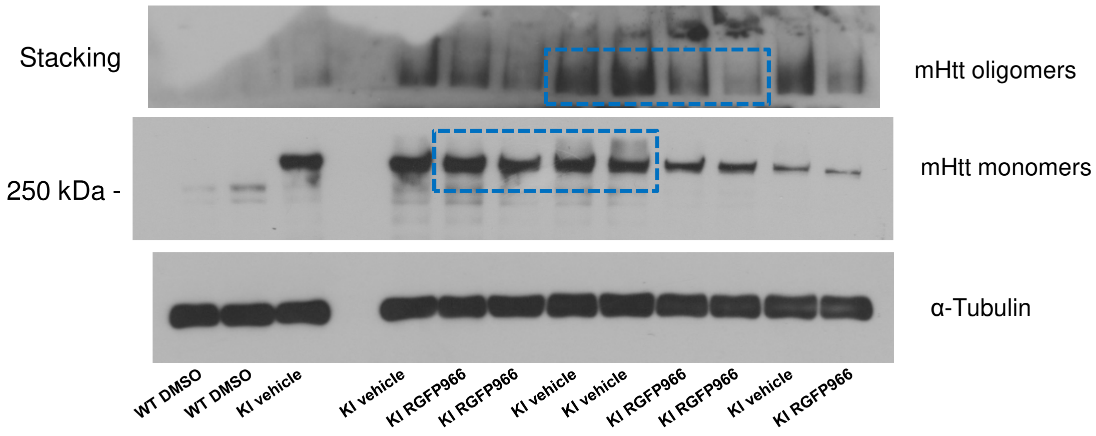

Supplementary Figure 5: original images of cropped immunoblots in Figure 7

Supplementary Figure 1a

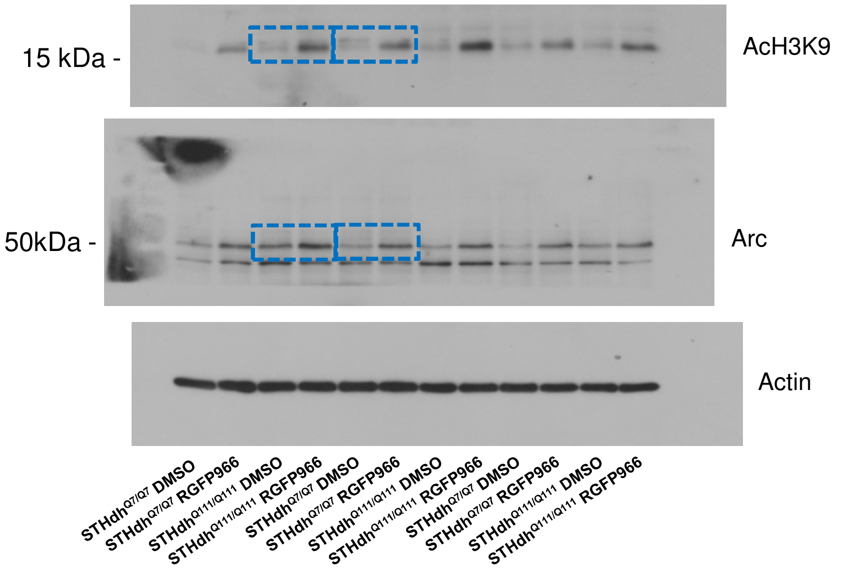

Supplementary Figure 1b

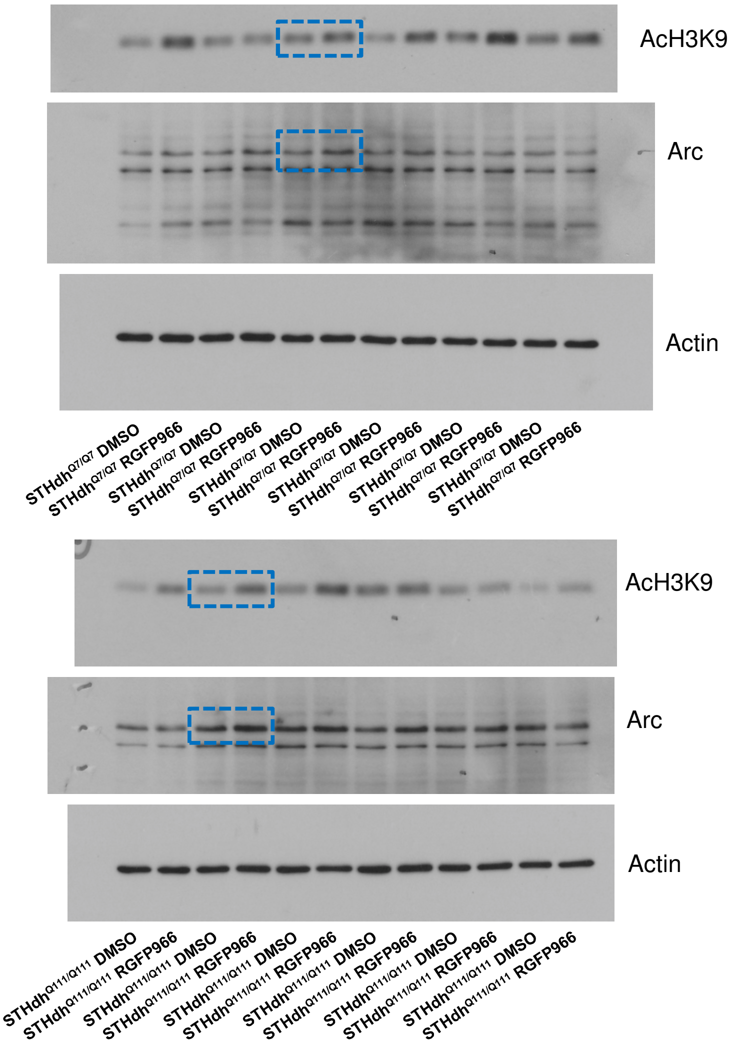

Supplementary Figure 6: original images of cropped immunoblots in Supplementary Figure 1
